# Optogenetics: Control of Brain Using Light

**Published:** 2018-01

**Authors:** Hamid Gholami Pourbadie, Mohammad Sayyah

**Affiliations:** Department of Physiology and Pharmacology, Pasteur Institute of Iran, Tehran, Iran

Neuronal cells communicate with each other by producing electrical signals or action potentials (APs). Different ion channels, including Na^+^, K^+^ and Ca^2+^ channels, are involved in generation of AP. Once an AP is generated in the soma, it travels down entire the axon length toward its terminal in a self-generating fashion that ultimately conveys information between neurons in the neural circuit. Depending on the neurotransmitter, each neuron inhibits or excites other neurons in a certain network. For instance, glutamate released from glutamatergic neurons, opens AMPA and NMDA channels permitting influx of Na^+^/Ca^2+^, which leads to postsynaptic depolarization. On the other hand, GABA released from GABAergic neurons results in Cl^-^ influx and postsynaptic hyperpolarization. One of the major challenges in neuroscience is how actions of individual cells in the brain could underlie a certain behavior such as attention, food consumption, aggression, cognition, and movement.

In 1979, Francis Crick suggested that controlling all cells from one type in the brain while leaving the others more or less unaltered is a real challenge for neuroscience. Electrical stimulation allows for temporal precision but it lacks spatial precision. In other words, it stimulates/inhibits all cell types (excitatory or inhibitory cells) located around the tip of the electrode. On the other hand, genetic and pharmacologic manipulations may have spatial precision but they are slow and lack temporal resolution on the timeframe of neural activity and signaling. Francis Crick speculated that a technology using light might be useful to control neuronal activity with temporal and spatial precision but at the time there was no technique to make neurons responsive to light.

Engineering neurons to express exogenous photo-sensitive proteins makes them responsive to light by alteration of membrane potential or signaling pathway. Several bacterial photo-sensitive proteins such as channelrhodopsin, halorhodopsin, and Jaws were used in optogenetic experiments. After introducing fiber-optic neural interfaces in 2007, studies of bacterial opsins were developed from cell culture to animal behavior, even in freely moving animals. Since then, application of optogenetics has been rapidly extended to investigate brain diseases, neural circuits, cancer, stem cells, urinary system, and vision, as well as cardiac and skeletal tissues. Such increasing importance of this technique in different fields of biomedical research led to it being chosen as the “method of the year” in 2010.

Generally, expression of light-sensitive opsins in the cell membrane induces alteration of membrane ion permeability in response to illumination. Channelrhodopsin-2 (ChR2), the opsin derived from *Chlamydomonas reinhardtii*, was the first photosensitive protein used to control neuronal activity. This opsin is a non-selective cationic channel that allows Na^+^ and Ca^2+^ influx in response to blue light. After exposure to blue light, the channel immediately depolarizes the neuron and then generates APs. Expression of ChR2 can be restricted to specific types of neurons by injection of viral vectors encoding the opsin to specific brain areas or by incorporating the opsin under the control of a specific promoter activated in the same types of neurons. Using genetically and/or anatomical approaches, many studies restrict expression of the opsin to specific brain regions or cell types of interest. For example, an AAV construct encoding Cre-dependent ChR2-EYFP was delivered into dorsomedial striatum of transgenic mice expressing Cre recombinase under the control of regulatory elements for the dopamine D1 or D2 receptor on the medium spiny neurons in the direct and indirect pathway of basal ganglia. Illumination of ChR2 at the direct pathway ameliorated Parkinsonian motor deficits in a mouse model of Parkinson’s disease. On the other hand, stimulation of indirect pathway in intact animals causes appearance of symptoms of Parkinson’s disease. Similarly, Tsai *et al.*, delivered Cre activated AAV carrying Chr2-EYFP into the ventral tegmental area (VTA) of tyrosine hydroxylase-IRES–Cre transgenic mice, thereby restricted expression of the opsin to dopaminergic neurons. They show that phasic but not tunic stimulation of dopaminergic neurons in VTA resulted in behavioral conditioning.

A different set of opsins is used for suppression of neuronal activity. Halorhodopsin derived from the species *Natronomonas pharaonis* (NpHR) is a photo-sensitive inwards chloride pump. It mediates chloride ions influx inducing 40-100 pA hyperpolarizing currents in response to yellow light illumination, which is sufficient for neural silencing. The eNpHR3 is a latest optimized version of NpHR. Using CaMKII IIα promoter associated with NpHR3 as an inhibitory opsin is strategy to inhibit specifically pyramidal cells in the brain. Photo- stimulation of pyramidal neurons lead to inhibition of excitatory neurons which may finally dampen seizure attacks in in animal models of epilepsy. Light-activated outwards proton pumps, archaerhodopsins, are also used for neural silencing. Arch, ArchT, and Mac, which are derived from *Halorubrum sodomense*, Halorubrum genus, and fungus *Leptosphaeria maculans*, respectively are three main archaerhodopsins. Their activation results in efflux of protons that induces hyperpolarization and finally neuronal activity suppression. Recently, a new version of halorhodopsin, Jaws, has been developed. This opsin is a red-shifted cruxhalorhodopsin from *Haloarcula salinarum* that generates robust neuronal inhibition. Less absorbed by hemoglobin makes the red light applicable to stimulate cortical neurons up to 3 mm deep from skull surface.

**Fig. 1:**
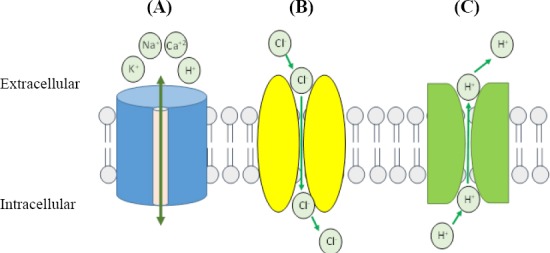
Common opsins used in optogenetics. (A) Channelrhodopsins passively permit Na^+^, K^+^, H^+^, and Ca^2+^ transport down their electrochemical gradients and depolarize neurons. (B) Halorhodopsins hyperpolarize neurons by actively pumping Cl^−^ against electrochemical gradients into the cell. (C) Archaerhodopsins hyperpolarize the neurons by actively transport of H^+^ out of the neuron.

## Potential clinical application of optogenetics

In addition to experimental use, optogenetics may have clinical applications. It has been shown that restricting ChR2 expression into second-order neurons (ON bipolar cells) in a mouse model of retinal degeneration makes them responsive to light and induces light-evoked potentials in both retinal ganglion and visual cortex. These findings provide the possibility for optogenetics to be used for treatment of people suffering from congenital or acquired blindness. Another possible application of optogenetics is in treatment of lower urinary tract dysfunctions such as overactive bladder or detrusor under-activity disorder by restricted expression of ChR2 or NpHR in smooth muscle cells. Drug resistant epilepsy is another disorder, which may be controlled by optogenetics in which excitatory and inhibitory networks involved in seizures, could be controlled by NpHR and ChR2, respectively.

**More details in:**

***The development and application of optogenetics*.** L Fenno et al. 2011. Annu Rev Neurosci; Vol. 34, pp. 389-412.

***Optogenetics: Novel Tools for Controlling Mammalian Cell Functions with Light*.** T Kushibiki et al. 2014. Int J Photoenerg; 895039: 10 pages.

***Regulation of parkinsonian motor behaviours by optogenetic control of basal ganglia circuitry*.** AV Kravitz et al. 2010. Nature; Vol. 466, pp. 622-626.

***Phasic firing in dopaminergic neurons is sufficient for behavioral conditioning*.** HC Tsai et al. 2009. Science; Vol. 324, pp. 1080-1084.

***Genetically encoded molecular tools for light-driven silencing of targeted neurons*.** BY Chow et al. 2012. Prog Brain Res; Vol. 196, pp. 49-61.

***On-demand optogenetic control of spontaneous seizures in temporal lobe epilepsy*.** E Krook-Magnuson et al. 2013. Nat Commun; Vol. 4, pp.1-8.

***Noninvasive optical inhibition with a red-shifted microbial rhodopsin*.** AS Chuong et al. 2014. Nat Neurosci; Vol. 17. pp. 1123-1129.

***Light-activated channels targeted to ON bipolar cells restore visual function in retinal degeneration*.** PS Lagali et al. 2008. Nat Neurosci; Vol. 11. pp. 667-675.

